# Development of a Radiologic Nomogram to Predict Invasiveness in Pulmonary Pure Ground-Glass Opacities: Analysis of the GORDON Cohort

**DOI:** 10.3390/cancers18111737

**Published:** 2026-05-26

**Authors:** Chiara Catelli, Susanna Guerrini, Miriana D’Alessandro, Sofia Lo Conte, Maria Antonietta Mazzei, Alfonso Fiorelli, Lorenzo Rosso, Mario Nosotti, Giuseppe Marulli, Andrea Dell’Amore, Stefano Margaritora, Beatrice Leonardi, Debora Brascia, Federico Rea, Andrea Lloret Madrid, Chiara Giraudo, Rossella Reale, Giampiero Dolci, Vincenzo Ambrogi, Federico Mathieu, Alexandro Patirelis, Maria Teresa Congedo, Filippo Lococo, Luca Luzzi, The Gordon Study Group

**Affiliations:** 1 Thoracic Surgery and Lung Transplant Unit, Department of Medicine, Surgery and Neurosciences, Azienda Ospedaliero-Universitaria Senese, University of Siena, 53100 Siena, Italy; lloretandrea@gmail.com (A.L.M.); mathieufede@gmail.com (F.M.); luca.luzzi@unisi.it (L.L.); 2 Diagnostic Imaging Unit, Department of Medicine, Surgery and Neurosciences, Azienda Ospedaliero-Universitaria Senese, University of Siena, 53100 Siena, Italy; guerrinisus@gmail.com (S.G.); mariaantonietta.mazzei@unisi.it (M.A.M.); 3 Department of Life Sciences, Health and Healthcare Professions, Link Campus University, 00165 Rome, Italy; dalessandromiriana@gmail.com; 4 Unit of Diagnostic and Therapeutic Neuroradiology, Department of Neurology and Human Movement Sciences, Azienda Ospedaliero-Universitaria Senese, 53100 Siena, Italy; sofia.loconte@student.unisi.it; 5 Thoracic Surgery Unit, University of Campania Luigi Vanvitelli, 81100 Naples, Italy; alfonso.fiorelli@unicampania.it (A.F.); beatrice.leonardi@unicampania.it (B.L.); 6 Thoracic Surgery and Lung Transplant Unit, Fondazione IRCCS Ca’ Granda-Ospedale Maggiore Policlinico, Department of Pathophysiology and Transplantation, University of Milan, 20122 Milan, Italy; lorenzo.rosso@unimi.it (L.R.); mario.nosotti@unimi.it (M.N.); 7 Department of Biomedical Sciences, Humanitas University, 20089 Milan, Italy; giuseppe.marulli@hunimed.eu (G.M.); deborabrascia@gmail.com (D.B.); 8 Division of Thoracic Surgery, IRCCS Humanitas Research Hospital, Humanitas University, 20089 Milan, Italy; 9 Division of Thoracic Surgery, Department of Cardiac, Thoracic, Vascular Sciences and Public Health, University of Padua, 35128 Padua, Italy; dellamore76@libero.it (A.D.); chirtoracicasiena@gmail.com (F.R.); 10 Department of Thoracic Surgery, Fondazione Policlinico Universitario A. Gemelli-IRCCS, Università Cattolica del Sacro Cuore, 00168 Rome, Italy; stefano.margaritora@policlinicogemelli.it (S.M.); mariateresa.congedo@policlinicogemelli.it (M.T.C.); filippo.lococo@policlinicogemelli.it (F.L.); 11 Unit of Advanced Clinical and Translational Imaging, Department of Cardiac, Thoracic, Vascular Sciences and Public Health, University of Padua, 35128 Padua, Italy; chiara.giraudo@unipd.it; 12 Department of Thoracic Surgery, Azienda Ospedaliera-Universitaria Sant’Anna, 44124 Ferrara, Italy; rss.reale@gmail.com (R.R.); giampiero.dolci@ospfe.it (G.D.); 13 Unit of Thoracic Surgery, Department of Surgical Sciences, Tor Vergata University Polyclinic, 00133 Rome, Italy; vincenzo.ambrogi@uniroma2.it (V.A.); alexandro.patirelis@hotmail.it (A.P.)

**Keywords:** ground-glass opacity, lung adenocarcinoma, computed tomography, nomogram, invasiveness, NSCLC

## Abstract

Background: Most predictive models for assessing the invasiveness of pure ground-glass nodules (pGGOs) have been developed in Asian populations, which may limit their applicability to Western cohorts. As the detection of pGGOs continues to increase, there is a growing need for reliable, population-specific tools to support preoperative decision-making. Methods: This multicenter retrospective study analyzed patients from the GORDON database who underwent surgical resection for pGGOs < 40 mm between January 2013 and June 2024. Radiologic features were assessed using preoperative high-resolution and contrast-enhanced CT scans. Univariate and multivariable logistic regression analyses were performed to identify independent predictors of invasive adenocarcinoma (IAC). A radiologic nomogram was developed and internally validated using a training (80%) and validation (20%) cohort. Results: A total of 490 pGGOs were included, of which 421 (85.9%) were IAC and 69 (14.1%) noninvasive (Adenocarcinoma in Situ or Minimally Invasive Adenocarcinoma). Upon multivariable analysis, maximum radiologic diameter (adjusted odds ratio [aOR] = 1.09, *p* = 0.001), spiculated margins (aOR = 3.07, *p* = 0.006), and unenhanced CT attenuation (aOR = 1.01, *p* < 0.001) were independent predictors of invasiveness. These variables were incorporated into a nomogram demonstrating good discrimination, with an area under the curve (AUC) of 0.86 (95% CI, 0.81–0.90) in the training cohort and 0.80 (95% CI, 0.70–0.90) in the validation cohort. Conclusions: A radiologic nomogram based on routinely available CT features enables accurate estimation of invasive adenocarcinoma risk in pGGOs. By integrating parameters beyond lesion size, this tool supports personalized management and may improve preoperative decision-making.

## Simple Summary

The increasing use of low-dose CT scans for lung cancer screening has led to the detection of many small lung nodules appearing as pure ground-glass nodules. While many of these lesions grow slowly and may not require immediate treatment, some already represent invasive cancer. Distinguishing between these forms before surgery is challenging and often leads to uncertainty in clinical decision-making. In this study, we developed a predictive tool based on common CT scan features to estimate the likelihood that a nodule is invasive. Using data from multiple centers, we created a visual model and an online calculator to support clinicians in assessing individual risk. This approach may help guide treatment decisions, reduce unnecessary surgery for low-risk lesions, and improve the selection of patients who would benefit most from early intervention.

## 1. Introduction

Pulmonary pure ground-glass opacities (pGGOs) represent a heterogeneous entity with a wide spectrum of biological behavior, and their preoperative characterization remains a major challenge in thoracic oncology despite advances in high-resolution computed tomography (HRCT). At the same time, the widespread adoption of low-dose computed tomography (LDCT) screening programs has led to an increased detection of pulmonary nodules, including a growing proportion of pGGOs [1]. pGGOs are often biologically indolent, with slow growth and favorable prognosis [2,3]. The pathological spectrum of pGGOs ranges from preinvasive lesions (adenocarcinoma in situ, AIS) to minimally invasive adenocarcinoma (MIA) and invasive adenocarcinoma (IAC). Although many pGGOs are indolent, a significant proportion may already represent IACs. In clinical practice, the management of pGGOs remains highly variable. Some lesions are followed with imaging surveillance, while others are referred for surgical resection without preoperative histological confirmation. This variability reflects the lack of reliable tools for preoperative risk stratification, as current guidelines are largely based on nodule size and density and are not specifically designed to discriminate invasive from preinvasive lesions within pGGOs [4]. Several predictive models and nomograms have been proposed to estimate the probability of invasiveness in lung adenocarcinoma presenting as pGGOs [5,6,7]. However, these models have been almost exclusively developed in Asian populations, where the epidemiological, clinical, and biological characteristics of lung cancer differ significantly from those observed in Western cohorts. In particular, Asian studies report a higher prevalence of pGGOs in younger, predominantly female, and often non-smoking patients, with a different distribution of histological subtypes and molecular profiles. For example, in a cohort of surgically resected solitary GGNs, more than two-thirds of lesions were IAC, with a predominance of female non-smokers [8]. These population-specific characteristics, together with differences in screening strategies and surgical indications, may substantially influence the performance and generalizability of predictive models. Consequently, the applicability of these nomograms to Caucasian populations remains uncertain, as Western patients typically present with different risk factor profiles, including a higher prevalence of smoking-related disease, distinct tumor biology, and potentially different imaging phenotypes. The GORDON database [9] represents the first large-scale dataset specifically focused on pGGOs in a Caucasian population, providing a unique opportunity to investigate imaging and clinical predictors of invasiveness in this setting. The aim of this study was therefore to develop a qualitative–quantitative imaging nomogram tailored to a European population, capable of estimating the individual probability of invasive adenocarcinoma in patients presenting with pGGOs.

## 2. Materials and Methods

### 2.1. Study Population

This multicenter retrospective study included all patients enrolled in the GORDON (Ground-glass Opacities Retrospective Database for Oncological N-status) database, comprising patients with pulmonary adenocarcinoma presenting as pGGO with a maximum diameter <40 mm who underwent surgical resection consecutively between January 2013 and June 2024. According to the IASLC/ATS/ERS classification of lung adenocarcinoma, pGGO was defined as a focal, homogeneous increase in lung attenuation on CT that preserved the visibility of underlying broncho-vascular structures and lacked any measurable solid component on both lung and mediastinal window settings [10]. Inclusion criteria required histopathologic confirmation of lung adenocarcinoma, availability of preoperative HRCT and contrast-enhanced CT (CECT) scans, and complete clinical and follow-up information. Patients with a history of prior lung cancer, primary lung tumors other than adenocarcinoma, or pulmonary metastases were excluded from the analysis. All included patients underwent at least two preoperative CT scans prior to surgical resection. All included pGGOs were either radiologically stable or demonstrated interval increase in size over time. No lesions showing radiologic regression were included in the analysis. The study was conducted in accordance with the Declaration of Helsinki and ethical review and approval were waived due to the retrospective observational design of the study and the use of fully anonymized data collected during routine clinical practice. No study-specific interventions or modifications of patient management were performed. All patients provided written informed consent for the use of their clinical data for research purposes. We used the Standards for Reporting of Diagnostic Accuracy Studies (STARD) checklist when writing our report. Data were collected from 8 Italian high-volume oncologic centers. Histopathologic evaluation of surgically resected pGGOs was considered the reference standard for this diagnostic accuracy analysis. For each lesion, histologic subtype and pathologic tumor size were documented. Final diagnoses were established according to recognized classification criteria, categorizing lesions based on the degree of invasiveness into AIS, MIA, and IAC. [10]. For analytical purposes, lesions were further grouped into two categories: invasive (IAC) and non-invasive (AIS and MIA, non-IAC). Postoperative surveillance consisted of thoracoabdominal computed tomography performed every 6 months during the first 2 years, followed by annual imaging thereafter.

### 2.2. Imaging Evaluation

**Figure 1:**
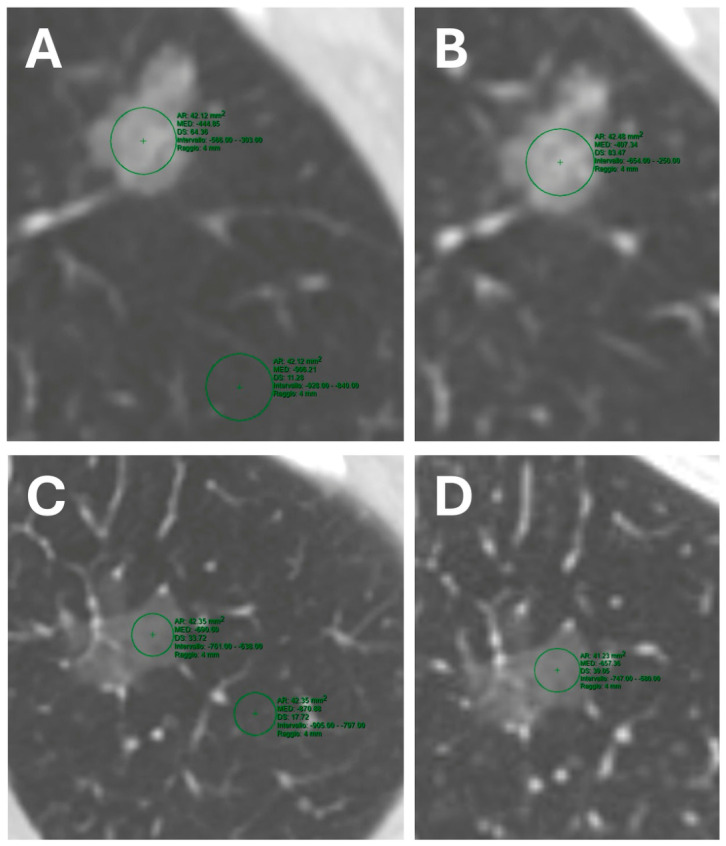
Pure ground-glass opacity (pGGO) and healthy lung density measurements on preoperative computed tomography (CT) scan, from which the “difference CT value” and “relative attenuation” are derived. The images shown are from the same patient, who presented with two synchronous pGGOs. (**A**) Unenhanced CT of an invasive adenocarcinoma (IAC). Two 4-mm-radius regions of interest were placed on the tumor (−444.85 HU) and on adjacent healthy lung parenchyma at least 10 mm away (−906.21 HU). (**B**) Post-contrast arterial-phase CT acquisition of the same lesion, here displaying a higher density (−407.34 HU). (**C**) Unenhanced CT of an adenocarcinoma in situ (AIS). The tumoral tissue density is −690.60 HU, lower than that of the other nodule, while the surrounding healthy tissue density is −870.88 HU. (**D**) The same AIS, in the post-contrast CT acquisition, shows an increased attenuation of −657.36 HU.

All imaging predictors were derived from preoperative HRCT and contrast-enhanced computed tomography (CECT) examinations performed at the participating institutions. Imaging was acquired with patients in the supine position using multidetector CT systems, predominantly 64-detector row scanners. All examinations included volumetric helical acquisition with thin-section reconstruction (<1 mm) suitable for multiplanar evaluation and quantitative attenuation analysis. Acquisition parameters were based on institutional thoracic imaging protocols and included tube voltages ranging from 100 to 120 kVp with automatic or weight-adapted tube current modulation, depending on scanner manufacturer and patient characteristics. Pitch values varied between 0.8 and 1.5 according to scanner generation and institutional protocols. Images were reconstructed using high-spatial-frequency lung kernels for parenchymal analysis and reviewed using standardized lung window settings (window width: 1500 HU; window level: −500 HU). Contrast-enhanced CT examinations were performed after intravenous administration of iodinated contrast material through peripheral venous access using power injectors. Image acquisition was obtained during the late arterial phase, approximately 45–50 s after contrast administration, according to local institutional protocols. Injection rates ranged between 2.5 and 4 mL/s and were followed by saline flush administration. All CT images were independently reviewed by two experienced readers at each participating center (one thoracic radiologist and one thoracic surgeon), both blinded to histopathologic outcomes. In cases of discrepant interpretation, consensus was reached through joint image re-evaluation before final data collection. Image interpretation was performed on dedicated workstations using multiplanar reconstructions. Candidate predictors included both qualitative and quantitative CT features selected a priori based on clinical relevance and existing literature. Qualitative variables comprised lesion location, margin characteristics (round or spiculated), pleural retraction, bubble-like lucency, bronchus sign, and vascular relationships (arterial versus venous). In cases of dual vascular supply, the predominant (largest) vessel was considered. Quantitative variables included maximum radiologic diameter, GGO attenuation on unenhanced and contrast-enhanced scans, and derived attenuation metrics. Lesion size was defined as the maximum diameter measured on axial images and confirmed on multiplanar reconstructions. CT attenuation measurements were obtained using a region of interest (ROI) placed within the most representative portion of the lesion while carefully avoiding visible vessels and bronchi. The same ROI was subsequently copied onto contrast-enhanced images to ensure measurement consistency. Reference lung parenchyma attenuation was measured on unenhanced images using an ROI positioned in normal lung tissue at least 10 mm away from the lesion. Derived variables included the difference in CT attenuation (GGO attenuation minus parenchymal attenuation) and relative attenuation (ratio of parenchymal to GGO attenuation). The quantitative analysis method is illustrated in Figure 1.

All predictor definitions and measurement methods are summarized in Table 1.

### 2.3. Statistical Analysis

Descriptive statistics were estimated. Categorical variables were reported as absolute frequencies and percentages, while quantitative variables were summarized using mean and standard deviation or median and interquartile range (IQR), according to the normal distribution assessed using the Shapiro–Wilk test. Comparisons between IAC and non-IAC patients were performed using Student’s t- test or the Mann–Whitney U test for continuous variables, depending on data distribution, and the Chi-square test or Fisher’s exact test for categorical variables, as appropriate. Univariate receiver operating characteristic (ROC) curves were constructed and the area under the curve (AUC) with 95% confidence intervals (CI) was calculated. Missing data were imputed using the median for quantitative variables and the mode for categorical variables, stratified by sex and histology (IAC, MIA, or AIS). The dataset was then split into a training sample (80%) and a validation sample (20%), maintaining the proportion of IAC of the original dataset. A stepwise logistic regression model was performed in the training sample to discriminate IAC from non-IAC. Finally, a nomogram was constructed based on the stepwise logistic regression model. Model performance was assessed using ROC curves and AUC with 95% CI in both the training and validation sets. A cut-off point with 90% sensitivity was selected in the training set and then applied to the validation cohort. *p*-value < 0.05 was considered statistically significant. All analyses were performed using R software version 4.5.2.

## 3. Results

### 3.1. Patients’ and Nodules’ Characteristics

A total of 490 pGGOs were included in the analysis, of which 421 (85.9%) were classified as IAC and 69 (14.1%) as non-IAC. Patient demographics and radiologic characteristics of the nodules, stratified on invasivity, are presented in Table 2.

There were no significant differences between the two groups in terms of age (median 69 vs. 67 years, *p* = 0.329) or sex distribution (male: 48.0% vs. 46.4%; *p* = 0.907). The median time from radiologic diagnosis to surgery was 10 months (IQR: 4–23), reflecting the frequent adoption of an initial radiologic surveillance strategy before surgical indication. Lesion location did not significantly differ between groups (*p* = 0.222). IAC had a significantly larger maximum diameter (median 21 vs. 15 mm, *p* < 0.001). Round margins were more commonly observed in non-IACs (76.5% vs. 53.6%; *p* = 0.001), whereas pleural tags were significantly more frequent in IACs (68.1% vs. 47.1%; *p* = 0.001). Bronchus sign was also more prevalent in IACs (46.7% vs. 27.9%; *p* = 0.006). The presence of bubble-like lucency (26.4% vs. 20.9%; *p* = 0.417) and the type of vascularization (arterial vs. venous; *p* = 0.547) did not significantly differ between groups. IACs showed higher (less negative) attenuation values on both unenhanced CT (median −355 vs. −550 HU; *p* < 0.001) and arterial-phase CT (median −405 HU vs. −564 HU; *p* < 0.001). CT attenuation was significantly greater in IACs (median −489 vs. −315 HU; *p* < 0.001), as was the relative attenuation (median 2.24 vs. 1.61; *p* < 0.001).

### 3.2. Radiologic Nomogram for Prediction of Invasive Adenocarcinoma

Univariate logistic regression analysis identified several radiologic variables significantly associated with invasive adenocarcinoma (Table 3).

**Figure 2:**
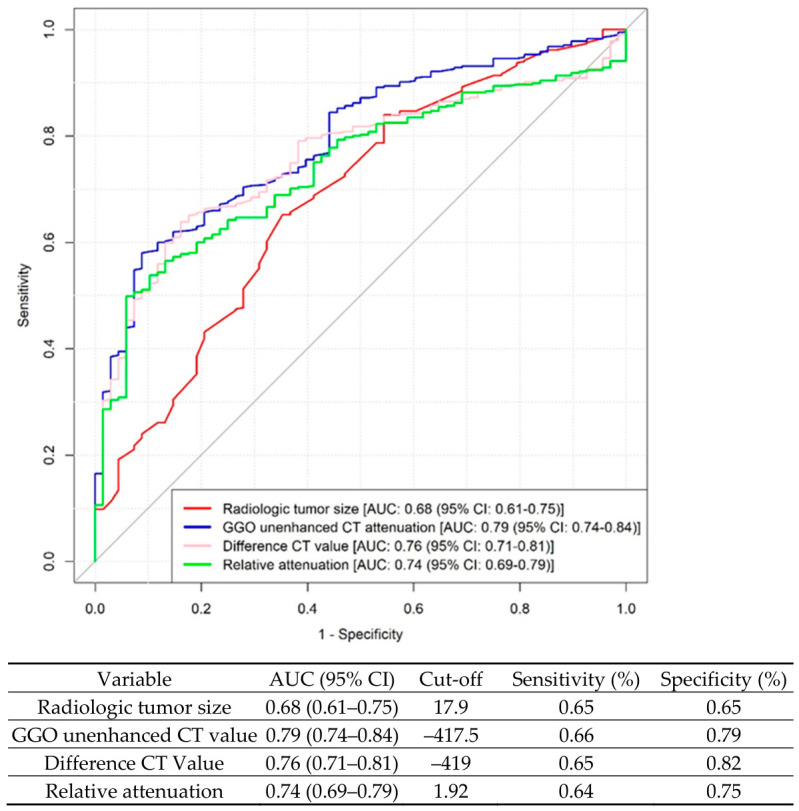
Univariate ROC Curve Analysis of quantitative radiologic predictors of Invasive Adenocarcinoma in Pure Ground-Glass Nodules.

Maximum radiologic diameter (OR = 1.10, *p* < 0.001), spiculated margins (OR = 3.44, 95%, *p* < 0.001), pleural tag (OR = 2.74, *p* < 0.001), bronchus sign (OR = 2.41, *p* = 0.006), and unenhanced GGO attenuation (OR = 1.01, *p* < 0.001) were all significantly associated with invasiveness. Difference in CT attenuation was also significant upon univariate analysis (*p* < 0.001), whereas relative attenuation was not (*p* = 0.73). Receiver operating characteristic (ROC) curve analysis was performed on quantitative CT-derived variables to identify predictors of invasiveness in pGGOs (Figure 2).

**Figure 3:**
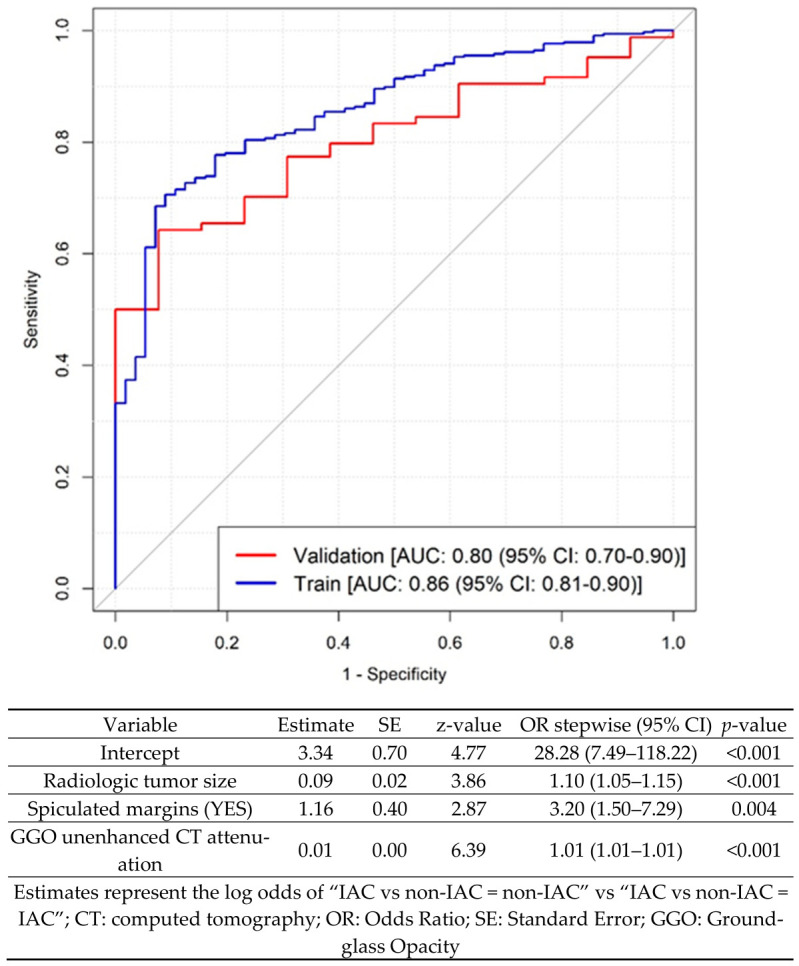
Receiver Operating Characteristic (ROC) Curve of the Nomogram for Predicting Invasive Adenocarcinoma in Pure Ground-Glass Nodules. The ROC curves demonstrate the discriminative performance of the radiologic nomogram in both the training and validation cohorts. The model achieved an area under the curve (AUC) of 0.86 (95% CI, 0.81–0.90) in the training set and 0.80 (95% CI, 0.70–0.90) in the validation set, indicating good predictive accuracy and satisfactory generalizability.

After stepwise multivariable analysis, three radiologic variables emerged as independent predictors of IAC and were incorporated into the final nomogram (Figure 3): maximum radiologic diameter (aOR = 1.09, *p* = 0.001), spiculated margins (aOR = 3.07, *p* = 0.006), and higher unenhanced CT attenuation values (aOR = 1.01, *p* < 0.001). Among qualitative features, spiculated margins represented the strongest predictor of invasiveness, with more than a threefold increased likelihood of IAC compared with round lesions.

Given their independent predictive value, these variables were incorporated into the development of the radiologic nomogram. The model demonstrated strong discriminative performance, with an area under the curve (AUC) of 0.86 (95% CI, 0.81–0.90) in the training cohort and 0.80 (95% CI, 0.70–0.90) in the validation cohort, indicating satisfactory generalizability (Figure 2). A cut-off value of 0.696 was selected. Using this value, the model yielded a sensitivity of 89.9%, a specificity of 50.0%, and an accuracy of 84.2% (95% CI: 80.2–87.7%) in the training set. In the validation cohort, the same threshold resulted in a sensitivity of 86.9%, a specificity of 38.5%, and an overall accuracy of 80.4% (95% CI: 71.1–87.8%).

**Figure 4:**
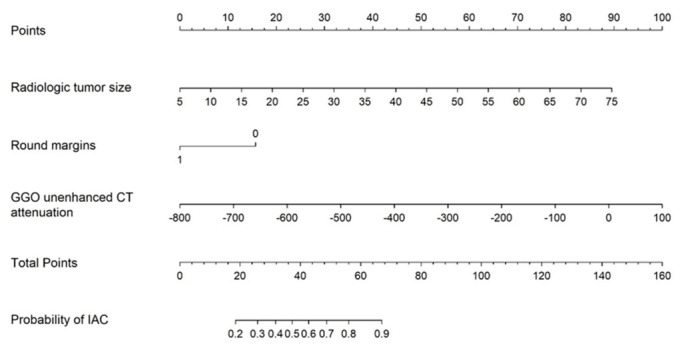
Clinical–Radiologic Nomogram for Predicting Invasive Adenocarcinoma in Pure Ground-Glass Nodules. The nomogram provides a graphical tool to estimate the probability of invasive adenocarcinoma. For each predictor, a corresponding point value is assigned by projecting vertically to the “Points” scale. The individual points are then summed to obtain a total score, which is subsequently projected onto the probability scale to determine the estimated risk of invasiveness. Nodule diameter is expressed in millimeters (mm), and unenhanced CT attenuation is reported in Hounsfield units (HU). Margin characteristics are entered as a binary variable, with a value of 1 for round margins and 0 for spiculated margins.

Based on multivariable analysis, a point-based radiologic nomogram was developed to easily estimate the individual probability of IAC in patients with pGGO (Figure 4).

**Figure 5:**
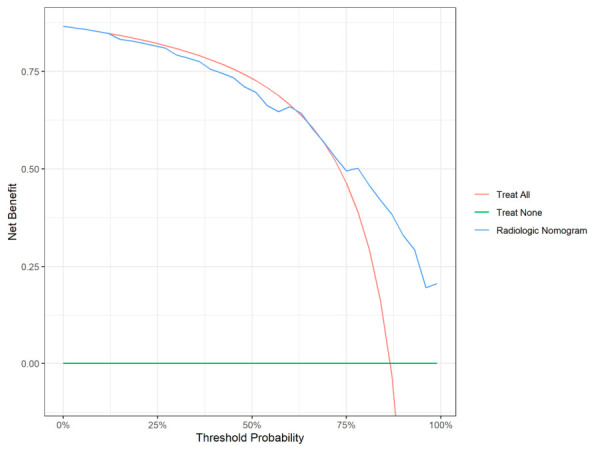
Decision Curve Analysis of the Radiologic Nomogram.

Each variable was assigned a weighted score proportional to its relative contribution to the model, allowing calculation of a total score corresponding to an individualized risk of invasiveness. The nomogram provides a graphical representation of the predictive model, enabling straightforward bedside application and facilitating individualized risk stratification. An online calculator was also developed and is available at the following website: https://gordoncalculator.shinyapps.io/calcolatore-iac-eng/, accessed on 26 March 2026. Decision Curve Analysis (Figure 5) confirmed the clinical utility of the nomogram, demonstrating that the model effectively supports surgical decision-making and helps in optimizing the management of pure GGOs.

## 4. Discussion

The increasing implementation of lung cancer screening programs has led to a rising detection of pGGOs, posing significant challenges in clinical management. Although these lesions are often associated with indolent biology, a substantial proportion may already harbor invasive adenocarcinoma. The high prevalence of IAC observed in our cohort aligns with recent data from Western populations and appears higher than that reported in Asian series, highlighting potential geographic and epidemiologic differences, but also reflects the different management of these lesions [11,12]. Importantly, the three independent predictors identified in the present study likely reflect distinct biological aspects of tumor aggressiveness. Increasing lesion size may indicate progressive neoplastic growth and greater invasive potential [13,14], while spiculated margins probably reflect stromal infiltration and desmoplastic reaction, both recognized hallmarks of invasive behavior [15]. Similarly, higher unenhanced CT attenuation values may reflect increased cellular density and reduced aerated components within the lesion, suggesting replacement of normal alveolar architecture by invasive tumor cells [16]. Taken together, these findings support the concept that radiologic morphology may represent a noninvasive surrogate of tumor biology in pGGOs. Notably, other features such as pleural tag and bronchus sign, although significant upon univariate analysis, did not retain independent predictive value, suggesting that their contribution may be mediated by other correlated variables. Current guidelines for the management of GGO generally consider pGGOs smaller than 30 mm as low-risk lesions [17], often suggesting a conservative approach based primarily on lesion size. However, it is now well known that the growth of pGGOs may occur not only as an increase in diameter, but also as an increase in attenuation or the appearance of solid components. Therefore, relying solely on lesion size as a discriminating factor is now considered reductive. While previous studies, including randomized trials, have demonstrated excellent postoperative outcomes for small pGGOs [18], our results do not aim to challenge these observations. Rather, they underscore that even lesions traditionally considered low risk may harbor invasive adenocarcinoma. In this context, the clinical value of the proposed nomogram lies in its ability to move beyond a size-based approach and to incorporate multiple radiologic features that better reflect tumor biology. The model, based on three independent predictors (maximum radiologic diameter, margin characteristics, and unenhanced CT attenuation), demonstrated good discriminative performance, with robust results in both the training and validation cohorts. These findings support the role of quantitative and qualitative CT features as reliable, noninvasive markers of tumor invasiveness. This is particularly relevant for avoiding a uniform management strategy for all pGGOs and instead enabling a more tailored approach. Identifying invasive components preoperatively may have important implications for surgical planning, including the choice between limited resection and more extensive procedures, as well as for overall patient management [19]. In particular, the possibility of safely performing limited resections is highly advantageous in the setting of small pGGOs, especially in experienced centers where preoperative or intraoperative nodule localization techniques are routinely employed [20,21]. This approach may allow adequate oncologic treatment while preserving lung parenchyma and reducing surgical invasiveness.

Several prediction models and CT feature analyses have been proposed to assess the risk of malignancy in pulmonary nodules; however, most were designed to differentiate benign from malignant lesions rather than to identify invasive disease within pGGOs. [22,23]. Most currently available predictive models for pGGOs have been developed in Asian populations, which differ substantially from Western cohorts in terms of epidemiology, smoking exposure, molecular landscape, and clinical management strategies. In this context, one of the major strengths of the present study is the development of a radiologic nomogram specifically derived from a large Caucasian population. This aspect is particularly relevant given the increasing implementation of lung cancer screening programs in Europe and North America, where population characteristics may substantially differ from those represented in previously published Asian-based models [6,8,24]. Another important strength of the study is its multicenter design, including patients from eight Italian high-volume thoracic oncology centers. This collaborative structure increases the robustness and external validity of the findings by reducing the impact of single-center selection biases and reflecting real-world variability in clinical practice and imaging assessment across Western institutions. In addition to the graphical nomogram, we developed a fully accessible online calculator to facilitate real-time application in routine clinical practice. This tool may enhance usability during multidisciplinary discussions and support individualized management strategies for patients presenting with pGGOs. Finally, DCA demonstrated that the proposed nomogram provided a higher net benefit than the “treat-all” and “treat-none” strategies across most clinically relevant threshold probabilities. These findings support the potential clinical utility of the model in improving individualized decision-making for patients with pGGOs. Although the present model was intentionally based on routinely assessable CT features to maximize clinical applicability, more advanced imaging approaches such as radiomics [25,26,27], automated texture analysis [28], and artificial intelligence-based image analysis [29,30] may further improve prediction accuracy and reduce observer dependency. Integration of these methodologies with conventional radiologic assessment represents a promising direction for future studies on pGGOs.

This study has several limitations. First, its retrospective design may introduce selection bias, particularly given that only patients undergoing surgical resection were included. As a result, the study population may be enriched for lesions with higher clinical suspicion, potentially overestimating the prevalence of IAC. Second, despite the use of a validation cohort, external validation in independent datasets is still required to confirm the robustness and reproducibility of the model. Moreover, despite predefined minimum imaging requirements for inclusion, minor variations in CT acquisition parameters and reconstruction algorithms among participating centers may have influenced attenuation measurements and qualitative radiologic assessment of pGGOs. In addition, interobserver variability, although mitigated using experienced readers, cannot be completely excluded. Finally, the model is based exclusively on radiologic features and does not incorporate clinical, molecular, or radiomic data, which could further enhance predictive accuracy.

## 5. Conclusions

In conclusion, this study presents a clinical–radiologic nomogram capable of estimating the probability of invasive adenocarcinoma in patients with pGGOs, based on routinely available CT features. By integrating multiple radiologic parameters beyond lesion size alone, the proposed nomogram allows a more refined assessment of tumor biology and may contribute to more personalized management of pGGOs. Further prospective studies and external validation are warranted to confirm these findings and to explore the integration of additional biomarkers into predictive models.

## Figures and Tables

**Table 1:** Definitions and Measurement of Radiologic Variables.

Variable	Definition/Measurement Method
Location	Lung lobe (Right upper, middle or lower lobe; Left upper or lower lobe)
Margins	Categorized as round or spiculated
Pleural retraction	≥1 linear strands extending from the nodule to the pleural surface
Bubble-like lucency	Round/ovoid air-containing areas within the lesion
Bronchus sign	Bronchus entering or coursing within the nodule
Vascularization	Feeding vessel classified as arterial or venous; largest vessel used if dual supply
Nodule size	Maximum diameter measured on axial and multiplanar reconstructions
GGO attenuation (unenhanced)	ROI placed within lesion, avoiding vessels/bronchi (lung window)
GGO attenuation (contrast-enhanced)	Same ROI copied onto arterial-phase images
Parenchymal attenuation	ROI on lung parenchyma (≥10 mm from the lesion, lung window, unenhanced CT)
Difference CT value	GGO attenuation − lung parenchyma attenuation
Relative attenuation	Lung parenchyma attenuation/GGO attenuation

ROI: Region of Interest; GGO: Ground-glass Opacity; CT: Computed Tomography.

**Table 2:** Clinical and Radiologic Characteristics of pGGO According to Invasive Status.

	Total (*n* = 490)	IAC (*n* = 421)	Non-IAC (*n* = 69)	*p*
Age, y	69.00 [62.00, 74.00]	69.00 [62.00, 74.00]	67.00 [60.00, 74.00]	0.329
Sex = male, %	234 (47.8)	202 (48.0)	32 (46.4)	0.907
Location, %				0.222
RLL	81 (16.6)	65 (15.5)	16 (23.2)	
LLL	61 (12.5)	51 (12.1)	10 (14.5)	
RML	18 (3.7)	15 (3.6)	3 (4.3)	
RUL	194 (39.7)	175 (41.7)	19 (27.5)	
LUL	135 (27.6)	114 (27.1)	21 (30.4)	
Radiologic max diameter, mm	20.00 [15.00, 26.00]	21.00 [15.00, 27.00]	15.00 [11.88, 22.00]	**<0.001**
Round margins, %	277 (56.8)	225 (53.6)	52 (76.5)	**0.001**
Pleural tag, %	318 (65.2)	286 (68.1)	32 (47.1)	**0.001**
Bubble-like lucency, %	125 (25.7)	111 (26.4)	14 (20.9)	0.417
Bronchus sign, %	215 (44.1)	196 (46.7)	19 (27.9)	**0.006**
Arterial vascularization, %	280 (57.9)	244 (58.5)	36 (53.7)	0.547
GGO unenhanced CT attenuation, HU	−379.00 [−502.00, −259.00]	−355.00 [−472.00, −224.00]	−550.00 [−623.50, −430.25]	**<0.001**
GGO arterial CT attenuation, HU	−437.50 [−565.12, −272.00]	−405.00 [−531.50, −182.00]	−564.00 [−611.25, −440.00]	**<0.001**
Difference CT value, HU	−459.12 [−597.25, −331.00]	−489.00 [−619.00, −366.49]	−314.73 [−407.75, −241.00]	**<0.001**
Relative Attenuation	2.11 [1.62, 3.04]	2.24 [1.68, 3.24]	1.61 [1.39, 1.92]	**<0.001**
Pathologic tumor size, mm	16.00 [11.00, 22.00]	17.00 [12.00, 23.00]	10.00 [8.00, 15.00]	**<0.001**

Data are presented as median [interquartile range (IQR)] or number (%), as appropriate. IAC: invasive adenocarcinoma; AIS: adenocarcinoma in situ; MIA: minimally invasive adenocarcinoma; GGO = ground-glass opacity; CT = computed tomography; RUL = right upper lobe; RML = right middle lobe; RLL = right lower lobe; LUL = left upper lobe; LLL = left lower lobe.

**Table 3:** Univariate and Multivariable Logistic Regression Analysis of Radiologic Predictors of Invasive Adenocarcinoma.

	Univariate Logistic Regression Analysis				Multivariable Logistic Regression Analysis			
Variables	Estimate	SE	OR (95% CI)	*p*-Value	Estimate	SE	OR (95% CI)	*p*-Value
Radiologic max diameter	0.09	0.02	1.10 (1.05–1.15)	**<0.001**	0.09	0.03	1.09 (1.04–1.15)	**0.001**
Spiculated margins	1.24	0.35	3.44 (1.78–7.21)	**<0.001**	1.12	0.41	3.07 (1.42–7.18)	**0.006**
Pleural Tag	1.01	0.29	2.74 (1.55–4.90)	**<0.001**	0.16	0.35	1.17 (0.58–2.34)	0.65
Bronchus sign	0.88	0.32	2.41 (1.31–4.66)	**0.006**	0.35	0.39	1.42 (0.67–3.09)	0.36
GGO unenhanced CT attenuation	0.01	0.001	1.01 (1.01–1.01)	**<0.001**	0.01	0.002	1.01 (1.01–1.01)	**<0.001**
Difference CT Value	−0.002	0.00	1.00 (1.00–1.00)	**<0.001**	0.002	0.001	1.00 (1.00–1.00)	0.18
Relative attenuation	0.002	0.01	1.00 (0.99–1.02)	0.73	0.01	0.05	1.01 (0.95–1.05)	0.79

GGO: Ground-glass Opacity; CT: Computed Tomography; OR: Odds Ratio.

## Data Availability

The data underlying this study are derived from the GORDON (Ground-glass Opacities Retrospective Database for Oncological N-status) multicenter database and are not publicly available due to institutional and ethical restrictions. De-identified data may be made available by the corresponding author upon reasonable request and with permission from the participating centers.

## Abbreviations

The following abbreviations are used in this manuscript:

AIS	Adenocarcinoma in situ
AUC	Area under the curve
CECT	Contrast-enhanced computed tomography
CT	Computed tomography
GGO	Ground-glass opacity
HRCT	High-resolution computed tomography
IAC	Invasive adenocarcinoma
LDCT	Low-dose computed tomography
MIA	Minimally invasive adenocarcinoma
pGGO	Pure ground-glass opacity
ROI	Region of interest
